# Association of Serum MicroRNA Expression in Hepatocellular Carcinomas Treated with Transarterial Chemoembolization and Patient Survival

**DOI:** 10.1371/journal.pone.0109347

**Published:** 2014-10-02

**Authors:** Mei Liu, Jibing Liu, Liming Wang, Huiyong Wu, Changchun Zhou, Hongxia Zhu, Ningzhi Xu, Yinfa Xie

**Affiliations:** 1 Laboratory of Cell and Molecular Biology & State Key Laboratory of Molecular Oncology, Cancer Institute & Cancer Hospital, Chinese Academy of Medical Sciences & Peking Union Medical College, Beijing, PR China; 2 Department of Interventional Surgical Oncology, Cancer Hospital of Shandong Province, Shandong Academy of Medical Sciences, Jinan, Shandong, China; 3 Department of Abdominal Surgery, Cancer Institute & Cancer Hospital, Chinese Academy of Medical Sciences & Peking Union Medical College, Beijing, PR China; 4 Clinical Laboratory, Cancer Hospital of Shandong Province, Shandong Academy of Medical Sciences, Jinan, Shandong, China; Xiangya Hospital of Central South University, China

## Abstract

**Background and Aim:**

Hepatocellular carcinoma (HCC) is one of the most deadly tumors. Transarterial chemoembolization (TACE) is effective for unresectable HCC. In recent years, miRNAs have been proposed as novel diagnostic and prognostic tools for HCC. This study aimed to identify whether microRNAs (miRNAs) can serve as biomarkers to reliably predict outcome before HCC patients are treated with TACE.

**Methods:**

Eleven miRNAs (miR-, miR-19a, miR-101-3p, miR-199a-5p, miR-200a, miR-21, miR-214, miR-221, miR-222, miR-223 and miR-, -5p) were quantified by quantitative real-time PCR (qRT-PCR) in 136 HCC patients’ serum before they received TACE therapy. Univariate and multivariate analysis were used to identify the prognostic value of clinical parameters and miRNAs. Area under the receiver operating characteristic curve (AUC) was used to evaluate the prediction potency.

**Results:**

The levels of some miRNAs were dramatically associated with clinicopathologic features regarding Child-Puge class, AFP, tumor size and satellite nodules. Univariate analysis revealed that miR-200a, miR-21, miR-122 and miR-224-5p were significantly associated with patients’ survival. Multivariate analysis demonstrated that AFP, satellite nodules and miR-200a were the independent prognostic factors associated with survival in this cohort (p = 0.000, 0.001, 0.000, respectively). The probability of the prognostic accuracy of miR-200a was 81.64% (74.47% specificity and 88.76% sensitivity), which was higher than the classifier established by combination of AFP and satellite nodules (76.87% probability, 70.21% specificity and 69.66% sensitivity). Furthermore, the combination of AFP, satellite nodules and miR-200a demonstrated as a classifier for HCC prognosis, yielding a ROC curve area of 88.19% (93.62% specificity and 68.54% sensitivity).

**Conclusions:**

Our study indicated that serum miR-200a may prognosticate disease outcome in HCC patients with TACE therapy. Therefore, miR-200a can potentially guide individualized treatment for HCC patients with a high risk of TACE treatment failures.

## Introduction

Hepatocellular carcinoma (HCC) is the most common type of malignancy of liver cancer. An estimated 748,300 new liver cancer cases and 695,900 cancer deaths occurred worldwide. Half of these cases and deaths were estimated to occur in China [1]. A great many HCC patients diagnosed at advanced tumor stages when standard surgery is not operable. Transarterial chemoembolization (TACE) treatment represents a first-line noncurative therapy for HCC and has been thought to be effective in improving survival of HCC patients with good liver function [2]. Most HCC patients receive TACE treatment. However, clinical outcomes vary significantly and are difficult to predict. The lack of effective outcome prediction models makes it difficult to apply individualized treatment protocols to HCC patients. A biomarker to accurately predict disease outcome before TACE therapy would be important for the early identification of patients with a high risk of treatment failures. For the high-risk patients, modified therapy or adjuvant therapy may potentially be applied to improve their survival.

MicroRNA (miRNA) is a type of endogenous non-coding RNA (ncRNA). They are responsible for post-transcriptional regulation and participate in nearly all biological processes [3]. The use of miRNA as cancer biomarker is of particular interest because it could be detected in blood plasma or serum with high stability [4]. In recent years, the therapeutic potential of miRNAs in HCC has been reported in various studies [5]–[7]. miRNAs have been proposed as novel diagnostic tools for classification and prognostic stratification of HCC. In light of reports from independent studies, consistent deregulation of miR-122, miR-199a-5p, miR-221 and miR-21 appears to be particularly important in HCC [8]–[10].

In this study, we selected 11 miRNAs to further validate in 136 HCC patients’ serum. All serum samples were collected before the HCC patients had been treated with TACE. The 11 miRNAs were selected based on the mining of public literatures that have been reported by different study cohorts of liver disease [11]–[19]. They were miR-122, miR-199a-5p, miR-221, miR-21, miR-101-3p, miR-200a, miR-214, miR-222, miR-223, miR-19a and miR-224-5p. Our study suggested that serum miRNAs can be considered as useful biomarkers that could help to stratify the prognosis and monitor follow-up in TACE-treated HCC patients. And the classifier of serum miR-200a outperforms the classifier established by the combination of AFP and satellite nodules in predicting the prognosis of TACE-treated HCC.

## Materials and Methods

### Patients with HCC

From January 2010 to July 2012, a total of 136 unresectable HCC patients who underwent TACE for the first time at Cancer Hospital of Shandong Province were included in this study. HCC was diagnosed according to the NCCN (National Comprehensive Cancer Network) guidelines. Status with respect to hepatitis B virus (HBV) infection was determined on the basis of HBsAg, HBsAb, HBcAb, HBeAg and HBeAg using commercially available immunoassay kits (Roche Diagnostics, Germany). AFP levels were determined by immunoenzymatic chemiluminescence (Roche Diagnostics, Germany). Clinicopathologic informations of the patient were summarized in Table 1. All serum samples were collected before the patients had received TACE. All of the patients were followed-up until November 2013.

**Table 1:** Clinicopathologic features in 136 HCC patients treated with TACE.

Parameters	Patients with HCC (n = 136)
**Gender**	
Male	118(86.8%)
Female	18(13.2%)
**Age (years)**	
≤60	86(63.2%)
>60	50(36.8%)
**BCLC Stage**	
A	9(6.6%)
B	82(60.3%)
C	45(33.1%)
**Child-Puge Class**	
A	85(62.5%)
B	51(37.5%)
**HBV**	
Yes	129(94.9%)
No	7(5.1%)
**Tumor size**	
≦5 cm	52(38.2%)
>5 cm	84(61.8%)
**AFP(ng/ml)**	
<20	45(33.1%)
20–400	37(27.2%)
>400	54(39.7%)
**Statellite nodules**	
Present	54(39.7%)
Absent	82(60.3%)
**Relapse**	
Yes	15(11.0%)
No	121(89.0%)
**Tumor multiplicity**	
Present	36(26.5%)
Absent	100(73.5%)

This study was approved by the medical ethics committee of Cancer Hospital of Shandong Province, and all the participants signed written informed consent forms.

### Treatment of Transarterial chemoembolization

Selective angiography was performed to identify the major arterial supply to HCC. TACE was conducted using a mixture of adriamycin, lipiodol and contrast agent. The dose of adriamycin and lipiodol were dependent on the tumor size and vascularity with 20–50 mg of adriamycin and 5–20 ml lipiodol per session. Subsequently, embolization was performed using gelatin sponge particles after TACE, and occlusion of target vessels and absence of additional tumor blood supply was confirmed.

### RNA isolation and qRT-PCR assay

Eleven candidate miRNAs were characterized in the serum samples by using SYBR-based real-time PCR (Quantobio Technology, Beijing, China). The QuantoBio Total RNA Isolation Kit was used to isolate RNA from the serum samples. The miR-Quanto System is composed with three reaction steps to convert and quantify levels of miRNA expression. Firstly, a polyadenine tail was attached to miRNA at their 3′ end. This is followed by a retro-transcription step that converts miRNA into cDNA and attaches a universal DNA tag at the 5′ end of synthesized cDNA. After the first strands of cDNA synthesized, qPCR was performed by using the miRNA specific forward primer and a reverse universal primer mix. The data were normalized using the external controls Quanto EC1 and Quanto EC2 which were added when the samples were extracted. All of the reactions were performed according to the manufacturer’s instructions.

### Survival analysis

Univariate Cox proportional hazards regression analysis were done to evaluate the association of each miRNA or clinical parameters to overall patient survival. The p values were calculated using the Wald test. Multivariate Cox proportional hazards regression analysis were done to evaluate the independent prognostic value of the miRNA signature or clinical parameters. The Kaplan-Meier estimator was used to evaluate the median survival time of the OS that was based on miRNA expression signature or clinical parameters. The p value of the Kaplan-Meier analysis was calculated with the log-rank test. Overall survival was defined as the time interval from the date of the first treatment of TACE to death or censored on the last follow-up.

### Statistical analysis

SPSS16.0 software was used for the statistical analysis. Statistical descriptions were used to describe the clinical pathological features, and the t test (Student’s t test) or ANOVA (analysis of variance) was used to analyze the measurement data. The p value was bilaterally tested, and values less than 0.05 were regarded as statistically significant. Logistic regression analysis was performed to analyze various combinations of clinical parameters and miRNA. The receiver operating characteristic (ROC) curve and the area under the curve (AUC) were used to determine the feasibility. The Youden’s Index was used to identify the optimal cut-off point. As defined, the corresponding sensitivity and specificity was showed.

## Results

### Association of clinical parameters with overall survival of HCC treated with TACE

**Figure 1:**
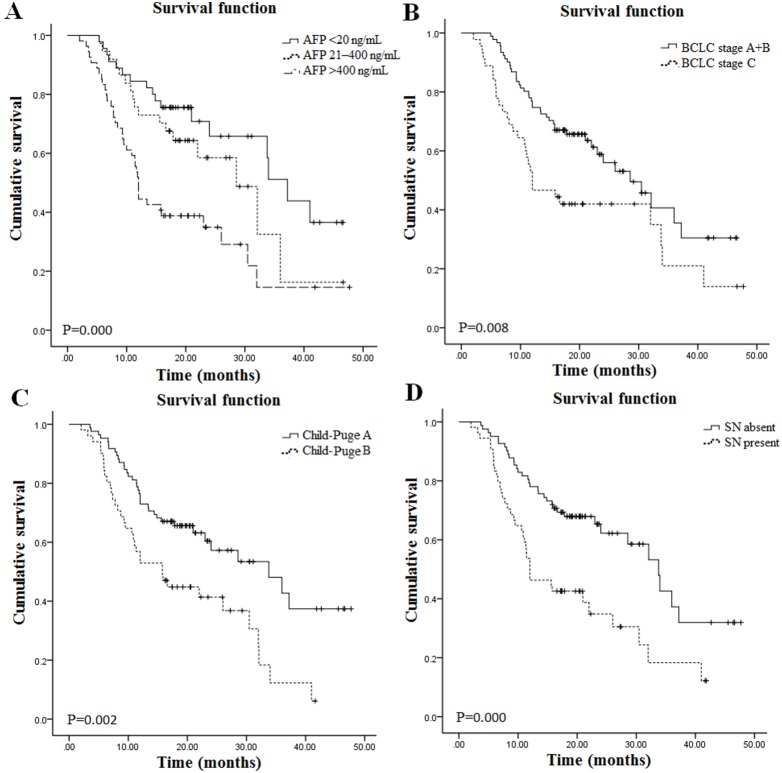
Association of clinical parameters with overall survival by Kaplan-Meier curves and the log-rank test. **A.** AFP-normal, AFP-elevated or AFP-diagnostic patients. **B.** Patients with BCLC stages A+B or C. **C.** Patients with Child-Puge class A or B. **D.** Patients with or without satellite nodules. SN: satellite nodules.

The characteristics of this patient cohort were summarized in Table 1. The prognostic values of multiple commonly used clinicopathologic features were analyzed with univariate analysis. AFP, BCLC stage, Child-Puge class and satellite nodules were significantly associated with survival (Figure 1 & Table 2), whereas other features, including gender, age at diagnosis, tumor size, HBV, relapse and tumor multiplicity, were not.

**Table 2:** Univariate and Multivariate Cox proportional hazards regression analysis of clinical parameters in relation to disease outcome.

Clinical variable	Overall survival			
	Univariate analysis		Multivariate analysis	
	HR(95%CI)	p value	HR(95%CI)	p value
BCLC Stage	1.871(1.167–3.000)	0.009		
Child-Puge Class	2.079(1.303–3.316)	0.002		
AFP	1.001(1.000–1.001)	0.000	1.001(1.000–1.001)	0.000
Satellite nodules	2.298(1.439–3.670)	0.000	2.240(1.403–3.578)	0.001

HR, hazard ratio; CI, confidence interval; BCLC, Barcelona Clinic Liver Cancer; AFP, alpha-fetoprotein.

According to the “diagnosis and treatment norms of primary hepatic carcinoma” that was issued by ministry of health of the PRC in 2011, AFP level of more than 400 ng/mL is a cut-off value to diagnose primary hepatic carcinoma. Here, HCC patients were divided into three AFP groups: normal (≤20 ng/mL), elevated (21–400 ng/mL), and diagnostic (>400 ng/mL). As shown in Figure 1A, for the AFP-normal HCC patients (n = 45), the median OS was 37.20 month, AFP-elevated HCC patients (n = 37), the median OS was 28.57 month, and AFP-diagnostic HCC patients (n = 54), the median OS was 12.00 month. The OS of the AFP-normal patients was statically longer than AFP-elevated and AFP-diagnostic HCC patients (p = 0.000). The group of patients with BCLC stage A and B had a longer OS than the group with BCLC stage C, the median OS was 28.56 month and 12.00 month, respectively (p = 0.008) (Figure 1B). The median OS of patients with Child-Puge A as 33.77 month and the median OS of patients with Child-Puge B was 15.77 month (p = 0.002) (Figure 1C). The group of patients with satellite nodules had a lower median OS (12.00 month) than the group without satellite nodules (33.77 month) (p = 0.000) (Figure 1D).

Multivariate Cox proportional hazard regression analysis revealed that AFP and satellite nodules were the independent prognostic factors associated with survival (Table 2).

### The expression of serum miRNAs was associated with HCC survival treated with TACE

**Figure 2:**
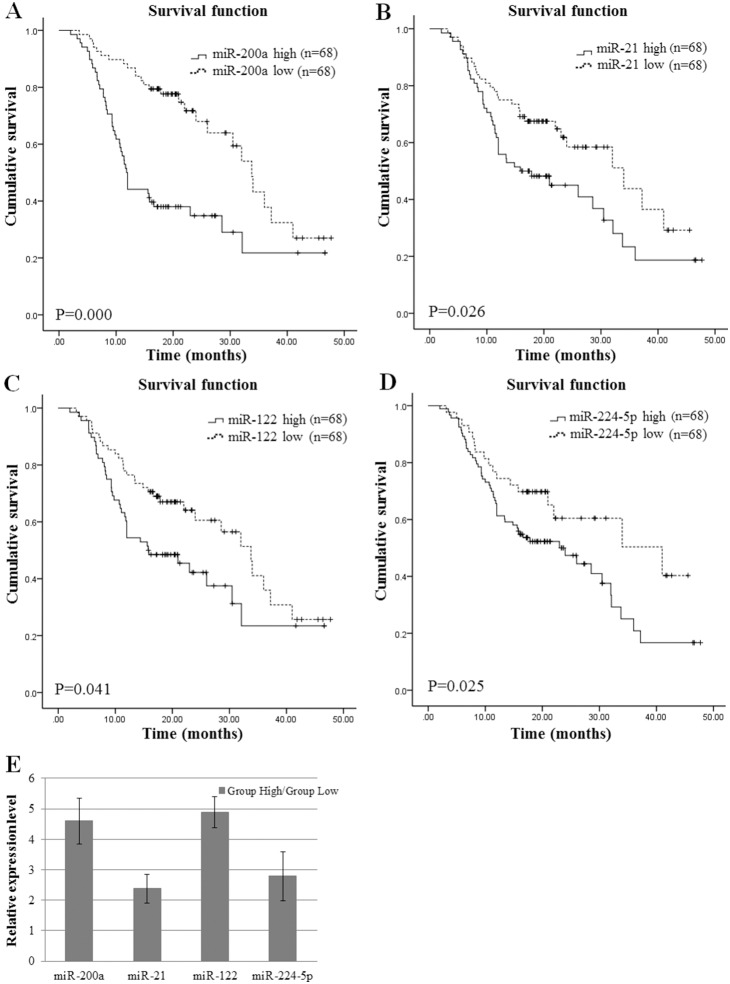
The levels of serum miRNAs were associated with overall survival. **A.** Patients with low or high expression of miR-200a (Cutoff value: Delta CT = 16.02). **B.** Patients with low or high expression of miR-21 (Cutoff value: Delta CT = 6.08). **C.** Patients with low or high expression of miR-122 (Cutoff value: Delta CT = 6.88). **D.** Patients with low or high expression of miR-224-5p (Cutoff value: Delta CT = 14.22). **E.** The relative expression level of serum miR-200a, miR-21, miR-122 and miR-224-5p in the group of HCC patients with high expression level was normalized by the group of patients with low expression level (set as 1), respectively. Mean ± s.d.

Eleven miRNAs were analyzed in 136 HCC patients’ serum to identify prognostic factors for outcome. By summarizing available data from independent studies, the selected miRNAs may have potential prognostic significance. The expression of individual miRNAs was correlated to overall patient survival with univariate analysis. We divided the 136 patients into two groups based on the median value of the expression level of each miRNA. Among the 11 miRNAs, four miRNAs (miR-200a, miR-21, miR-122 and miR-224-5p) were significantly associated with cancer survival, while the OSs of other groups were not significantly different (p>0.05). The Kaplan-Meier curves of OS according miR-200a, miR-21, miR-122 and miR-224-5p were plotted in Figure 2A–D. Comparing each miRNA expression in HCC serum with patient’s survival time revealed two group: those with predominantly higher expression of miR-200a, miR-21, miR-122, miR-224-5p and poor survival and those with predominantly lower expression of miR-200a, miR-21, miR-122, miR-224-5p and good survival (p = 0.000, 0.026, 0.025, 0.041, respectively). The relative expression level of each miRNA between the high and low group was showed in Figure 2E. The Multivariate Cox proportional hazard regression analysis revealed that miR-200a was the independent prognostic factor associated with survival (Table 3).

**Table 3:** Univariate and Multivariate Cox proportional hazards regression analysis of miRNAs in relation to HCC outcome.

Variable	Overall survival			
	Univariate analysis		Multivariate analysis	
	HR(95%CI)	p value	HR(95%CI)	p value
miR-200a	0.572(0.474–0.690)	0.000	0.572(0.474–0.690)	0.000
miR-21	0.700(0.528–0.926)	0.013		
miR-224-5p	0.700(0.553–0.886)	0.003		
miR-122	0.804(0.686–0.942)	0.007		

HR, hazard ratio; CI, confidence interval; miR, microRNA.

To further understand the significance of miRNAs in the prognosis of TACE-treated HCC patients, whether the 11 miRNAs expression were significantly associated with the clinicopathologic features were analyzed. As shown in table 4, univariate analysis showed that the expression of miR-101-3p, miR-199a-5p and miR-221 were considerably associated with Child-Puge stage, miR-21, miR-222 and miR-224-5p were associated with tumor size, miR-122, miR-200a, miR-214, miR-21 and miR-224-5p were associated with AFP, and miR-101-3p, miR-19a and miR-222 were associated with satellite nodules (p<0.05).

**Table 4:** Correlation between the expression of 11 miRNAs and clinical parameters of 136 HCC patients with TACE treatment.

Parameters	miRNAs	P value
Gender	None	
Age	None	
BCLC stage	None	
	miR-101-3p	0.026
Child-Puge class	miR-199a-5p	0.004
	miR-221	0.036
HBV	None	
	miR-21	0.018
Tumor size	miR-222	0.044
	miR-224-5p	0.046
	miR-122	0.017
	miR-200a	0.005
AFP	miR-214	0.022
	miR-21	0.023
	miR-224-5p	0.000
	miR-101-3p	0.005
Satellite nodules	miR-19a	0.010
	miR-222	0.039
Relapse	None	
Tumor multiplicity	None	

### The classifiers for predicting prognosis of hepatocellular carcinoma with TACE treatment

**Figure 3:**
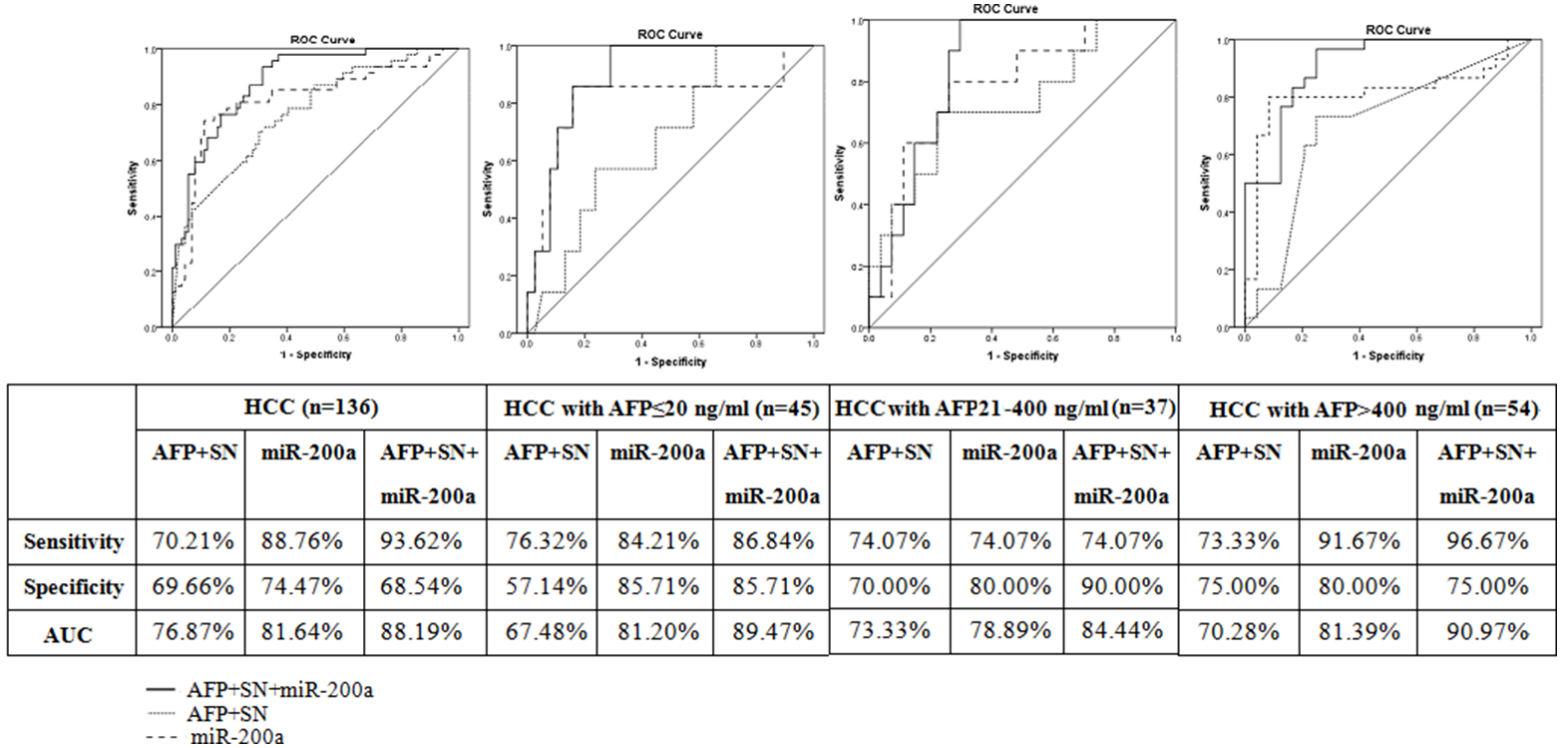
Receiver operating characteristic curve analysis for predicting prognostic accuracy of hepatocellular carcinoma with TACE treatment. ROC curves for the combination of AFP and satellite nodules, miR-200a and the combination of AFP, satellite nodules and miR-200a in total 136 patients and subgroup of HCC patients with normal (≤20 ng/ml), elevated (20–400 ng/ml) and diagnostic (>400 ng/ml) AFP level, respectively. The sensitivity, specificity and AUC were indicated below each ROC graph. SN: satellite nodules.

Multivariate analysis revealed that AFP, satellite nodules and miR-200a were the independent predict factor associated with patient survival. Then, the discriminative power of these factors in predicting the outcome before TACE-treatment to HCC patients was verified. According to the OS, the patients were stratified into two subgroups, including a sensitive group (>12 months) and a resistant group (≤12 months). To evaluate the diagnostic value, the ROC curve was used to analyze the sensitivity and specificity. As shown in Figure 3, the ROC curve of the combination of AFP and satellite nodules showed an AUC of 76.87% (69.66% sensitivity and 70.21% specificity) (Figure 3A). The ROC curve of miR-200a had an AUC of 81.64% (88.76% sensitivity and 74.47% specificity). Meanwhile, the combination of AFP, satellite nodules and miR-200a, yielding an AUC of 88.19% (68.54% sensitivity and 93.62% specificity), was proved to be a powerful discrimination tool.

These classifiers were tested in the subgroups of patients with normal-, elevated- and diagnostic-AFP, respectively. As shown in Figure 3, the AUC of miR-200a was 81.20% (sensitivity = 84.21%, specificity = 85.71%) in the normal AFP (≤20 ng/ml) group. In the elevated AFP (21–400 ng/mL) group, the AUC of miR-200a was 78.89% (sensitivity = 74.07%, specificity = 80.00%). In diagnostic (>400 ng/mL) group, the AUC of miR-200a was 81.39% (sensitivity = 91.67%, specificity = 80.00%). The analysis demonstrated that the miR-200a classifier was more powerful than the combination of AFP and satellite nodules in all three subgroups, especially in the normal AFP one.

## Discussion

In recent years, the clinical value of miRNAs has been assessed because of its tumor-specific expression and stability in tissues and in the circulation. Although miRNA expression signatures have been applied to the outcome prediction of HCC, no study to date has been reported for the application of miRNAs to patients with HCC that received TACE treatment. TACE is recommended as a single treatment modality in unresectable HCC. There are a few predictors of OS, including AFP and BCLC stage [20], [21], which are consistent with our study. Previous studies have reported the prognostic effect of AFP status after TACE and the dynamic change after locoregional therapy, including TACE [21], [22]. However, the prognostic significance of baseline AFP levels before treatment in HCC patients has not been well clarified. Our study demonstrated the prognostic value of AFP in HCC patients before TACE treatment. Patients with high AFP expression display significantly lower overall survival. Meanwhile, univariate analysis showed that the expression of miR-122, miR-214 and miR-200a were associated with AFP. It has been demonstrated that miR-122, a liver-specific miRNA, could regulate AFP via miR-122/CUX1/miR-214/ZBTB20 pathway [23]. A recent study showed that ASB4 is regulated by miR-200a directly in HCC and the level of ASB4 is associated with serum AFP [24]. These studies provide clues to support our results, although the exact molecular mechanisms that mediate AFP expression are needed to be elucidated.

When correlated the above 11 miRNAs with patient’s survival, we found that the higher presence of miR-200a, miR-21, miR-122 and miR-224-5p in HCC following TACE was associated with a decreased overall survival. The association at the tissue level between HCC and these four miRNAs has been previously reported. Huang et al demonstrated that miR-200a and miR-200b plays important roles in HCC migration by regulating E-cadherin expression [25]. Petrelli A et al showed that activation of the nuclear factor erythroid related factor 2 (NRF2) pathway and up-regulation of the miR-200 family were among the most prominent changes of early molecular changes in HCC. Further, miR-200a is known to negatively regulate the NRF2 pathway [26]. The findings of the above studies emphasized the important role of miR-200a in HCC. A recent study showed that miR-200a was significantly down-regulated in HCC tissue [27]. Our present study showed that HCC patients with higher serum miR-200a expression experienced worse survival. Thus, serum miR-200a maybe served as a marker to present disease severity-dependent change of HCC patients. Moreover, miR-21 has been reported as a potent oncogene that overexpressed in HCC and plays a key role in resisting programmed cell death in cancer cells [28], [29]. Consistent with the present findings, a newly published report suggested that circulating miR-21 has a prognostic value in patients with cancer [30]. miR-122 has been shown to be liver-specific and highly expressed in the normal liver. Previous reports showed that serum miR-122 may serve as a potential biomarker for liver injury [31]–[33]. Elevated miR-122 in the serum of patients may be released from the damage of hepatocytes caused by virus infection or cancer. This might explain why miR-122 is down-regulated in HCC tissue but elevated in serum of HCC patients. And, the change in miR-122 concentration appeared earlier than the increase in aminotransferase activity in the blood [31]. Li et al validated that miR-224 was overexpressed in HCC tissues and regulated cell migration and invasion by miR-224/HOXD10/p-PAK4/MMP-9 signaling pathway in HCC [34], [35]. These studies strongly support the importance of these four miRNAs in HCC development, although the roles of them need to be further studied.

In the present study, we have found that miR-200a was a robust classifier in predicting prognosis of HCC treated with TACE and outperformed the classifier of the combination of AFP and satellite nodules as biomarker with positive predictive value. When we combined AFP, satellite nodules and miR-200a together to predict the prognosis, the probability could be further improved (from 81.6% to 88.2%). Furthermore, the accuracy of the combination of AFP and satellite nodules in normal-AFP subgroup was 67.48%, lower than the 70% value that is considered highly useful. However, the classifier of miR-200a in normal-AFP subgroup presented an AUC of 81.20% with a sensitivity of 84.21% and a specificity of 85.71%, confirming the potential role of miR-200a in predicting the prognosis of HCC.

This study was a retrospective study with a relatively small sample size and the application of the classifier in TACE-treated HCC patients is yet to be further validated, but the aim is clearly different from previous. There has no study to date reporting the association of miRNA expression with HCC prognosis following TACE therapy. Our evidences highlight that serum miR-200a may be a promising prognostic biomarker in HCC patients. Patients with a high risk of TACE treatment failure may benefit from measurement of miR-200a in serum.

In summary, the AFP status, satellite nodules and miR-200a in serum before TACE may help us to predict patients’ survival, and it may also enables pretherapeutic stratification of HCC patients in designing a treatment strategy.
